# BEMOVI, software for extracting behavior and morphology from videos, illustrated with analyses of microbes

**DOI:** 10.1002/ece3.1529

**Published:** 2015-06-04

**Authors:** Frank Pennekamp, Nicolas Schtickzelle, Owen L Petchey

**Affiliations:** 1 Institute of Evolutionary Biology and Environmental Studies, University of ZurichWinterthurerstrasse 190, Zurich, CH-8057, Switzerland; 2 Earth & Life Institute, Université catholique de LouvainCroix du Sud 4, Louvain-la-Neuve, B-1348, Belgium; 3 Department of Aquatic Ecology, Eawag: Swiss Federal Institute of Aquatic Science and TechnologyÜberlandstrasse 133, Dübendorf, CH-8600, Switzerland

**Keywords:** Microbial ecology, microcosm, trait-based ecology, video analysis

## Abstract

Microbes are critical components of ecosystems and provide vital services (e.g., photosynthesis, decomposition, nutrient recycling). From the diverse roles microbes play in natural ecosystems, high levels of functional diversity result. Quantifying this diversity is challenging, because it is weakly associated with morphological differentiation. In addition, the small size of microbes hinders morphological and behavioral measurements at the individual level, as well as interactions between individuals. Advances in microbial community genetics and genomics, flow cytometry and digital analysis of still images are promising approaches. They miss out, however, on a very important aspect of populations and communities: the behavior of individuals. Video analysis complements these methods by providing in addition to abundance and trait measurements, detailed behavioral information, capturing dynamic processes such as movement, and hence has the potential to describe the interactions between individuals. We introduce BEMOVI, a package using the R and ImageJ software, to extract abundance, morphology, and movement data for tens to thousands of individuals in a video. Through a set of functions BEMOVI identifies individuals present in a video, reconstructs their movement trajectories through space and time, and merges this information into a single database. BEMOVI is a modular set of functions, which can be customized to allow for peculiarities of the videos to be analyzed, in terms of organisms features (e.g., morphology or movement) and how they can be distinguished from the background. We illustrate the validity and accuracy of the method with an example on experimental multispecies communities of aquatic protists. We show high correspondence between manual and automatic counts and illustrate how simultaneous time series of abundance, morphology, and behavior are obtained from BEMOVI. We further demonstrate how the trait data can be used with machine learning to automatically classify individuals into species and that information on movement behavior improves the predictive ability.

## Introduction

Microbes are crucial components of all ecosystems, providing important services such as organic matter decomposition, production of biomass and oxygen, or carbon storage (Kirchman 2012). Whereas tremendous progress has been made in describing the phenotype of microbes such as their physiology, other aspects of the phenotype such as behavior have progressed at a slower pace (Finlay 2004). Until recently, this was mainly due to methodological limitations which constrain descriptions of microbes to the population or community level (Kreft et al. 2013). While these approaches provided important insights into the functional diversity of microbes, limitations in describing microbes at the individual level would preclude understanding of ecological and evolutionary processes dependent on individual level characteristics, and intraspecific variability in such characteristics (Bolnick et al. 2003; DeAngelis and Mooij 2005). The same limitations apply to the use of microbial model systems such as protists, which have a long and successful tradition in testing ecological and evolutionary theory (Gause 1934; Elena and Lenski 2003; Holyoak and Lawler 2005; Kawecki et al. 2012; Altermatt et al. 2015).

Advances in microbiology have been driven by technological developments ever since Antonie van Leeuwenhoek invented the compound microscope (Kreft et al. 2013). New technologies such as metagenomic studies (Albertsen et al. 2013), flow cytometry (Müller and Nebe-von Caron 2010), digital analysis of still images (Schillinger et al. 2012), and single-cell microbiology (Brehm-Stecher and Johnson 2004) provide insights about the structure and composition of microbial communities, as well as the morphology, function and ecology of microbes at the individual level (Kreft et al. 2013). Whereas these new technologies are powerful in showing differences among individuals in physiology or morphology, they miss out on an important component of the individual phenotype: behavior. Behavior is important because it mediates the interaction strength in communities, for instance, between predators and prey (McGill and Mittelbach 2006) with effects on community properties such as stability (Fryxell and Lundberg 1998). Digital video analysis can complement these approaches by providing quantitative descriptions of behavior via automated tracking (Dell et al. 2014), which is collected at the individual level in addition to abundance and morphological data. Such methods apply to all types of empirical systems where individuals in the community are characterized by continuous movement, for example, they should work on samples taken in the field and also for micro- and mesocosm systems in laboratory conditions. Whereas a variety of commercial and open-source software exists to perform tracking (see supplementary material of Dell et al. 2014 for an overview), or to extract morphology and abundance data (e.g., DAIME, Schillinger et al. 2012), the combination of these capabilities is to our knowledge rare. Some software lacks efficient ways of dealing with large numbers of video files and may be difficult to customize and automate. Dell et al. (2014) give an extensive overview of tracking software and their strengths and weaknesses. While previous demonstrations relying on digital image analysis tested and validated work flows aimed at single-species microcosms (e.g., Mallard et al. 2013; Pennekamp and Schtickzelle 2013), extending the capability of such systems to more complex communities is required (Gaston and O’Neill 2004). Some success with automatic classification of species was achieved with protists in activated wastewater sludge (Amaral et al. 2008) and with automated systems to analyze abundance and quantify trait distributions during large-scale marine monitoring schemes (e.g., Zoo/PhytoImage: http://www.sciviews.org/zooimage/, Bell and Hopcroft 2008). These show that such efforts are worthwhile even with challenging field-collected samples. To our knowledge, no studies so far used automated video analysis in the context of automated species identification, although Branson et al. (2009) showed that it was possible to predict the gender and genotype of *Drosophila* flies from their dynamic movement behavior.

Automated video analysis usually consists of three steps: video acquisition, video processing/analysis, and data interpretation (Dell et al. 2014). To fulfill the latter two steps, we introduce a new R package, BEMOVI, and show its validity and scope of application. For guidance on the image acquisition step, refer to Dell et al. (2014) or Pennekamp and Schtickzelle (2013). BEMOVI is an automated digital video processing and analysis work flow to extract abundance, morphological, and movement data for tens to thousands of individuals in a video, hence characterizing a microbial population or community by multiple traits. We illustrate how this trait data can be used to predict species identity in a multispecies community, and how the characteristics of the movement improve the predictive ability of the classification model compared to morphological data only. We then derive population abundance by counting the individuals of each species and validate these against manual counts of a trained human observer taken simultaneously for both single and multispecies communities.

## Description of the BEMOVI Package and its Functions

The BEMOVI work flow relies on two freely available, open source, and cross-platform software widely used in the scientific community: R – the statistical computing environment (R Development Core Team 2012) and ImageJ, a powerful image processing and analysis software (Schneider et al. 2012). ImageJ shows considerably better performance than a native R solution for the video processing steps (Pennekamp and Schtickzelle 2013). We therefore built BEMOVI as a set of modular R functions (Table S1) calling ImageJ and reading its output, creating a seamless work flow that deals efficiently with large numbers of video files and merges results into databases for easy analysis. Additional helper functions are provided to help in setting up and validating the use of BEMOVI for a specific experimental system. BEMOVI is readily available from github (https://github.com/pennekampster/bemovi), a solution allowing for easy package upgrading, and has been thoroughly tested on Macintosh OS X, Windows 7 & 8, and Ubuntu Linux 14.04 LTS. Additional documentation and a full demonstration of the package can be found at www.bemovi.info.

BEMOVI is built to process a directory containing a set of video files shot with identical settings, with three main steps (Table1): (1) locate, measure, and reconstruct the movement trajectories of individuals in a video; (2) merge measurements from all treated videos into a single database to which information on experimental conditions is added; and (3) perform basic analyses and validating results.

**Table 1:** Overview of functions provided with the BEMOVI package. Functions are ordered according to the analysis flow

Step	Function name	Short description
Setup	check_video_file_names	Checks whether video files are either of ^*^.avi or ^*^.cxd format
	check_threshold_values	Assists finding manually an appropriate threshold for image segmentation
Locate, measure, and reconstruct trajectories	locate_and_measure_particles	Segments and thresholds video by difference image segmentation, then runs particle analysis to locate and measure the morphological properties of each particle on all frames
	link_particles	Reconstructs the movement trajectory of a given particle by linking its coordinates through time and calculates movement metrics such as turning angles and step lengths
Merge data	merge_data	Creates a database merging morphology and trajectory information with a description of the experimental design
Process data	summarize_trajectories	Summarizes the mean morphology and movement and its variability on the trajectory level
	filter_data	Filters the data, excluding very short, almost non-moving and low detection trajectories
Validation	create_overlays	Creates an overlay of the original video and the trajectories identified in the segmentation and tracking steps; two different visualization options are possible; if the species was predicted, trajectories can be colored according to the species they were predicted to belong to

### Identify, locate, and measure individuals

BEMOVI includes two functions: locate_and_measure_particles and link_particles. In the first, each single video is split into a stack of images (=frames) ordered in time (Dell et al. 2014), and each of these frames is treated sequentially to locate and measure individuals (function: locate_and_measure_particles). To discriminate individuals from a background, BEMOVI uses the dynamic difference image segmentation which is a variant of background subtraction (Pennekamp and Schtickzelle 2013). The difference in intensity (gray value) between the frame to be analyzed and a reference frame at a constant time offset (e.g., 25 frames or 1 second later) is calculated by subtracting the two frames from each other. The resulting difference image, which contains only the particles that moved (i.e., pixels that changed intensity), is then binarized (i.e., converted to black and white) using a user defined threshold. Both the time offset and the threshold must be carefully adjusted and validated by the user to minimize segmentation errors (e.g., immobile individuals are considered background and do not appear in the difference image, or the size and shape of individuals are biased due to partial overlap between the frames used for creating the difference image). Other segmentation approaches such as intensity thresholding or edge detection, which do not suffer from this problem, are compromised by the heterogeneous backgrounds and light conditions common in our experimental system (microbial microcosm communities) (Pennekamp and Schtickzelle 2013).

A helper function assists with finding the appropriate threshold (function: check_threshold_values). Binarized images are then analyzed by the ParticleAnalyzer tool of ImageJ (this is run automatically from R, within the function locate_and_measure_particles), which extracts for each particle, *X*- and *Y*-position and morphology (area, mean, minimum and maximum of the gray value, perimeter, width, length, and angle with the dominant axis of a fitted ellipse, circularity, aspect ratio, roundness, and solidity) (Schneider et al. 2012).

### Reconstruct movement trajectories

Reconstructing movement trajectories (with function link_particles) involves linking the position of each particle through the stack of images. The link_particles function in R uses the MOSAIC ParticleTracker plug-in for ImageJ (Sbalzarini and Koumoutsakos 2005), which performs both segmentation and tracking in its native form. However, the ParticleTracker also allows the input of *X*- and *Y*-coordinates themselves, having two advantages: (1) users can use whatever approach they want for segmentation and feed X- and Y-positions directly to the link_particle function, and (2) linking on X- and Y-positions only is more efficient as large variation in size and shape of the individual can hamper the detection in tracking applications (Dell et al. 2014).

Another useful feature of the ParticleTracker is its ability to track simultaneously hundreds of particles in an unrestricted viewing field (e.g., particles can move in and out), even when some of them miss out on certain frames due to detection problems. The algorithm deals with occlusions (i.e., when a particle collides with another particle, either individual or debris) conservatively by interrupting the current trajectory and starting a new one (Sbalzarini and Koumoutsakos 2005). So far, no attempts are made to recombine partial trajectories due to occlusions. Recombining tracks after occlusion often implies some assumptions on movements (e.g., it is more likely that individuals go straight so recombination is carried out such as minimizing turns at occlusion), but such assumptions might afterwards bias the analysis of movement.

Two arguments are required to parameterize the tracking function: The maximum possible displacement of particles between two successive frames and the number of frames over which a particle can be linked if missing on some intervening frame(s). They must be carefully validated to avoid errors (e.g., creating an erroneous link between different particles if displacement and/or link range are too large, or broken links if they are too small). After trajectories are reconstructed, movement metrics are computed for each pair of coordinates (between two frames) that form an individual trajectory: step length, absolute angle, turning angle, net squared displacement, and gross displacement. For a detailed description on the calculation and interpretation of these metrics, refer to textbooks on the quantitative analysis of trajectories such as Turchin (1998).

### Merge measurements from all treated videos into a single database

The second step (i.e., data merging) combines the morphology and movement metrics acquired on each video into a single database and links this information with a video description file containing any relevant information on experimental conditions for each video (e.g., treatment level, video capture settings), using the video file name for merging (function: merge_data).

### Perform basic analyses and validate results

In the third step (i.e., data processing), aggregation of the morphology and movement metrics (median, mean, and variability; see Table S1) is performed on the trajectory level (each trajectory is given a unique ID, that is, a combination of file name and trajectory number). This averaging over the trajectory will to a certain degree account for errors on cell morphology originating from imperfect segmentation such as described above (e.g., individuals partially overlap among the frames used to build the difference image). In case such problems occur more then only occasionally, this means that the processing has not been parametrized correctly and/or there is some intrinsic flaw in applying BEMOVI to that set of videos (e.g., because no reliable segmentation can be obtained). To validate the output of BEMOVI, we suggest using the create_overlays helper function for visualizing the extracted trajectories. This function overlays the original video data with the extracted trajectories, labelled with ID, and thus helps troubleshooting erroneous and incomplete tracking results. Videos in which individuals are not labelled with an ID (i.e., were not tracked), or whose label frequently changes, are indicators that the morphology extraction or the tracking parameters require fine-tuning. To distinguish individuals from remaining artefacts (e.g., moving debris), we provide another function to exclude particles that do not comply with certain minimum requirements, that is, minimum net displacement, trajectory duration, detection rate, and median step length (function: filter_data).

## Materials and Methods

### Species and experimental conditions

Microcosms of protists are widely used model systems in ecology and evolution (Altermatt et al. 2015). For the following experiments illustrating the use of the BEMOVI package, a set of nine small, single-celled ciliates was used: *Paramecium caudatum*, *Paramecium aurelia*, *Blepharisma japonicum*, *Colpidium striatum*, *Colpidium campylum*, *Cyclidium glaucoma*, *Tetrahymena thermophila*, *Didinium nasutum*, and *Loxocephalus* sp., which show variation in morphology (Giometto et al. 2013) as well as in movement behavior (Carrara et al. 2012).

The aquatic microcosms used followed a similar setup as Petchey (2000), and for detailed and comprehensive methods, please see Altermatt et al. (2015). For each experiment, ciliates were cultured in jars of 240 mL volume covered by an aluminum cap to allow air exchange but prevent contamination. Each jar contained 100 mL of bacterized medium before ciliates were added. The medium consisted of protist pellet medium (Carolina Biological Supplies, Burlington, NC) at a concentration of 0.55 g per liter of Chalkley’s medium, as well as two wheat seeds for slow nutrient release. The medium was then inoculated with three species of bacteria (*Serratia fonticola*, *Brevibacillus brevis* and *Bacillus subtilis*). Bacteria were cultured for a day at 37^°^C. Ciliates were then added to the jars and kept in temperature-controlled incubators at 20^°^C for the remainder of the experiments.

Three experiments were run to test and illustrate the performance and scope of the BEMOVI package:

1. Monocultures of eight ciliate species (previous list except for *Didinium nasutum*) were assembled, and videos taken to characterize the variation among species in terms of morphology and movement behavior.
2. Mono- and mixed cultures of *Colpidium striatum*, *Didinium nasutum*, *Paramecium caudatum*, and *Tetrahymena thermophila* were assembled and ten independent samples assessed by automatic and manual counts, in order to compare the abundances per experimental unit (the individual microcosm).
3. *Colpidium striatum*, *Paramecium caudatum*, and *Tetrahymena thermophila* were followed over 28 days in monocultures and mixed cultures by automatic and manual counts (nine sampling days), again to compare these two methods of observation.

### Microscope and video setup

All videos were taken using a stereomicroscope (Leica M205 C) with a 25× magnification mounted with a digital CMOS camera (Hamamatsu Orca C11440, Hamamatsu Photonics, Japan). Dark field illumination (LED ring light stage controlled by a Schott VisiLED MC 1500) was used such that the ciliates, usually transparent in bright field microscopy, appear white on black background; this greatly facilitates segmentation. For details how to setup the hardware for best segmentation results, please refer to Pennekamp and Schtickzelle (2013) or Dell et al. (2014).

To sample a culture, we transferred 1 mL of culture into a Sedgewick Rafter cell (S52, SPI supplies, Westchester, PA), which was placed under the microscope objective. We took videos at a frame rate of 25 frames per second in the proprietary .cxd format. The BEMOVI package is limited in the video formats and it can currently read (.avi and .cxd, via the BIO-formats plug-in), but could easily be extended to accommodate any of the many other video formats readable by ImageJ and the BIO-formats plugin (Linkert et al. 2010). As tracking parameters, we specified a link range of five frames for all three experiments and a displacement of 20 pixels for experiments 1 and 3. In experiment 2, a higher displacement of 25 pixels was required to account for the fast moving *Didinium nasutum*.

Trajectories were filtered by the filter_data function to get rid of artefacts such as spurious trajectories due to moving debris. Trajectories for analysis were required to show a minimum net displacement of at least 50 *μ*m, a duration of 0.2 sec, and a detection rate of 80% (for a trajectory with a duration of 10 frames, the individual has to be detected on at least eight frames) and a median step length of >2 *μ*m. In open systems where the viewing field is not restricted (i.e., individuals can swim in and out of the viewing field), automatic counts by BEMOVI are required on a by-frame basis and then averaged across the whole video. This avoids that occlusions resulting in multiple trajectories recorded for a single individual inflate the counts.

For manual counting, a sample was taken from the culture and manually counted under a dissecting microscope by an experienced experimenter (as described in Altermatt et al. 2015). Such manual observations, albeit very time-consuming and limited to abundance measurements, are still the most widely used method in experimental micro- and mesocosm studies (Pennekamp and Schtickzelle 2013). Therefore, we chose them as the standard against which to compare the automatic measurements by BEMOVI. However, as an alternative methodology, manually counting individuals on the recorded video would have the advantage of disentangling different sources of error (e.g., intersample variability, compensation between false positives and false negatives in the automatic segmentation procedure of BEMOVI). It was not chosen here for the following reasons: first, we consider the microcosm, not the sample, as the relevant experimental unit; second, a specific example may not be particularly informative because different sources of error are likely to vary widely among case studies as they are highly dependent on the video settings, the environment (amount of debris present) or the movement behavior of the target species; third, manual observation of videos, required for quantifying frequencies of different types of error, would be very cumbersome, and also prone to error.

As we considered the microcosm as the relevant experimental unit for all experiments, we took independent samples for manual and automatic counts; this has the drawback that samples were not paired, and thus, intersample variability may be confounded with tracking errors. However, the intersample variability also affects manual counting and should be overall comparable between methods as sampling is performed in a similar fashion.

### Machine learning for automated species identification

We used supervised machine learning to train and classify individuals in mixed cultures. The random forest (RF) classifier is a widely used classification algorithm based on ensembles of decision trees (Breiman 2001). By constraining the number of observations and variables included when constructing individual decision trees, trees within an ensemble are decorrelated and the identity assigned to an individual is usually based on the majority vote of the ensemble (Cutler et al. 2007). We trained the RF on the properties of individuals from monocultures and consequently used the model to predict species identity in mixed cultures using the same traits but on unidentified individuals. We report the classification success (1 - out-of-bag error rate; reported as percentage), which states how well the model performs on observations not included in training the model (i.e., a cross-validation).

## Results

**Figure 1:**
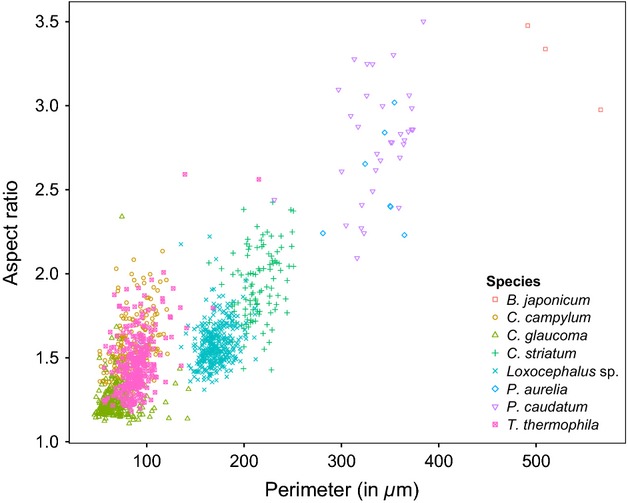
Morphological characteristics of eight ciliate species: cell perimeter and aspect ratio (major axis/minor axis of a fitted ellipse).

**Figure 2:**
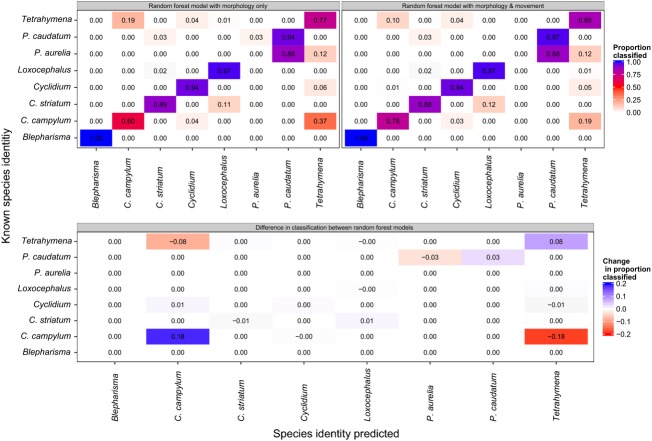
Confusion matrices between eight ciliate species to illustrate the improved classification success when both morphology and movement features are considered (>89%), compared to morphology only (84%). The lower panel shows the differences in classification success between the two models. Along the diagonal, increases in the proportion of correctly classified species are positive, indicating higher rates of successful classification, whereas off the diagonal, negative differences indicate decreasing classification error (fewer individuals are misclassified into an incorrect species).

Information on traits exhibited by individuals in a mixed community is usually used to predict their species identity as long as sufficient differences in trait space exist (Gorsky et al. 2010). Figure1 shows the differences in two morphological traits (cell perimeter and aspect ratio) among eight species grown separately in monocultures in experiment 1. Some species, such as *Paramecium caudatum* and *Paramecium aurelia*, show considerable morphological overlap, whereas many species occupy quite distinct areas of the morphological trait space, aiding in their automatic classification. Comparing manual cell length measurements collected from the literature and online sources with those measured by BEMOVI, we found a strong positive correlation (*R*^2^ = 0.85, *P* < 0.01, see Fig. S1), illustrating the correspondence between manual and automatic trait measurements. On average, we reached a classification success of 84% in our study; however, species overlapping strongly in trait space were impossible to distinguish, resulting in a complete failure to predict the identity correctly (see *P. caudatum* and *P. aurelia*). Figure2 shows species classification based on morphological traits, with higher misclassification of morphologically similar species, that is, 37% of known *Colpidium campylum* got identified as *Tetrahymena thermophila*, whereas 19% of known *T. thermophila* were misclassified as *C. campylum*. When the movement characteristics were considered in addition to morphology, classification success increased about 5% to an average of 89%, with a decrease in classification error by 18% in *Tetrahymena* and by 8% in *Colpidium* (Fig. 2). Highly similar species such as *P. caudatum* and *P. aurelia*, however, remain indistinguishable even after adding movement traits.

**Figure 3:**
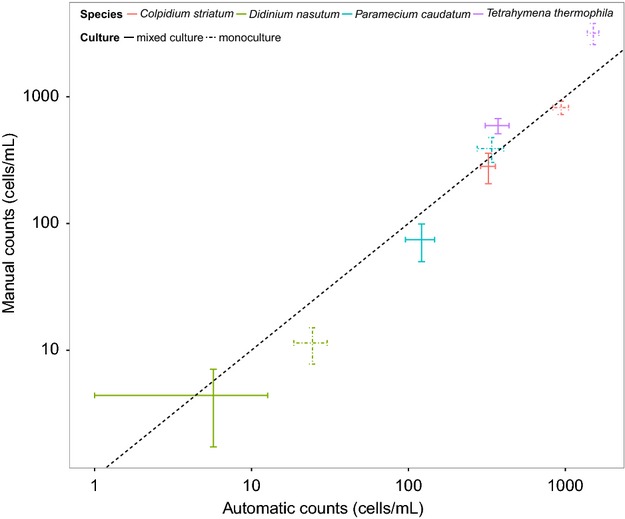
Comparison of manual and automatic estimates of population abundance in single (dashed bars) and mixed species cultures (solid bars). The ±1 STD error bars are calculated for the 10 repeated samples from each species and cultures (monocultures and mixed cultures) for manual and automatic counts. The dashed line indicates the 1:1 line. A barplot is used instead of showing the data point-by-point because the 10 samples for each treatment were not paired, that is, counting was not made on the same sample but rather on the same experimental unit (the microcosm).

In the second experiment, four ciliate species (*T. thermophila*, *P. caudatum*, *Colpidium striatum*, and *Didinium nasutum*) were grown in mono- and mixed cultures, with the aim to compare automatic (using BEMOVI) and manual counts. Because the four species used in this experiment are well separated in trait space, the out-of-bag classification error of the training model was <2%. We separately took 10 samples from each culture to assess the total amount of variability associated with sampling abundances. Automatic and manual counts showed high correspondence, regardless whether mono- and mixed cultures (containing all three ciliate species in the same microcosm) were considered (Fig. 3, *R*^2^: 0.94 and 0.94, mono- and mixed cultures, respectively). The among-sample variability was overall similar between the two counting methods.

**Figure 4:**
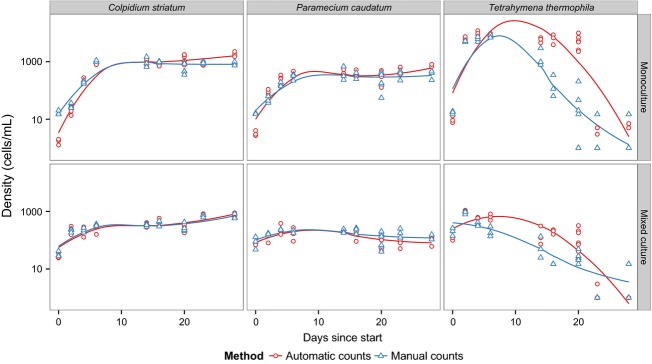
Population dynamics of three species of ciliates given by manual and automatic counting (fitted lines are local polynomial regressions) to illustrate the general growth dynamics. The mixed culture contains a combination of the three species used in the monocultures (*Colpidium striatum, Paramecium caudatum, Tetrahymena thermophila*).

**Figure 5:**
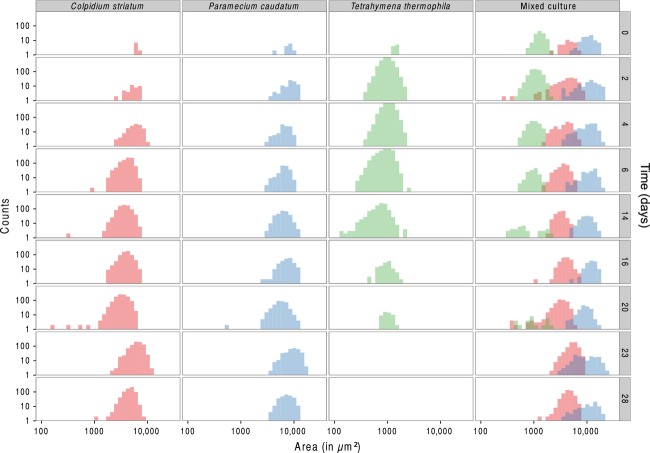
Body size changes through time (from first row to last row) in the mono- and mixed cultures during the experiment. At day 20, the medium was partly replaced by fresh medium perturbing the mono- and mixed cultures. Both axes are on log_10_ scaling.

**Figure 6:**
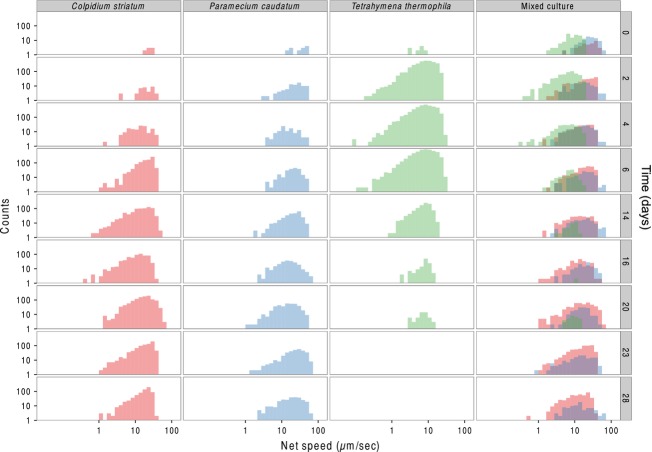
Changes in movement speed through time (from first row to last row) in the mono- and mixed cultures during the experiment. At day 20, the medium was partly replaced by fresh medium perturbing the mono- and mixed cultures. Both axes are on log_10_ scaling.

In experiment 3, we followed mono- and mixed cultures (containing all three species) for a period of 28 days and obtained species identification and estimation of abundance both automatically and manually. Both methods captured very well the monoculture growth dynamics of *P. caudatum*, *C. striatum*, and *T. thermophila* (Fig. 4). Although *T. thermophila* showed some discrepancy between the methods, overall both were closely correlated (*R*^2^: 0.86). Importantly, BEMOVI succeeded in automatically identifying the abundance of the three species when cultured together in a mixed culture, closely matching the counts of a trained human observer. Furthermore, cell size dynamics (Fig. 5) illustrate the ability of BEMOVI to capture trait dynamics. Over the first twenty days, *C. striatum* and *T. thermophila* decreased in size, whereas *P. caudatum* remained rather stable. After a resource pulse (replacement of 50% of the medium) on day 20, cell sizes increased. Figure6 shows how a dynamic trait such as movement speed changes during the 28 day experiment. The species show stronger overlap in movement speed than in size, but the distribution of speed seems more stable through time and unaffected by the resource pulse on day 20. Such patterns demonstrate the potential of BEMOVI in trait-based community ecology, although deep analysis of the patterns illustrated here is beyond the scope of this article.

## Discussion

Microbes are important for all the ecosystems on the planet, but are still mostly studied at the population or community level. This contrasts with the notion that ecological processes can be influenced by variation among individuals and that descriptions on the population or community level require incorporation of such differences (Bolnick et al. 2003; DeAngelis and Mooij 2005). Recent technological advances allow study of microbes at the individual level and discovery of whether variation among individuals is as important for microbes as for larger animals and plants (Kreft et al. 2013). The video analysis work flow we presented here complements techniques such as flow cytometry and metagenetic approaches, because it allows to study the behavior of microbes within populations and communities in situ. Characterizing behavior may thus reveal unrecognised functional diversity, previously masked by low morphological and potentially genetic differentiation.

BEMOVI shares the advantages of other automated image analysis systems: (1) results (videos) can be stored for later analysis or re-analysis, (2) use of a computer reduces observer bias, (3) video acquisition is usually faster than manual counts and the effort is constant regardless of community complexity, whereas manually counting complex communities can be very time-consuming (Pennekamp and Schtickzelle 2013). For illustration, an experiment including all pairwise combinations of six species required nine person-hours to count a total of 108 microcosms. In comparison, video acquisition was achieved in about 2.5 h and subsequent video processing by BEMOVI taking an additional 2 h. It is important to note, however, that setup and validation of such a system require initial time investment. Moreover, very complex videos, with many individuals (>1000), may also take considerable time to process (e.g., several hours for a single video). Still, given that nearly no intervention by humans is needed, the gains in real working time are considerable. In addition, the work flow presented here extracts far more information than manual observation because multiple traits are collected simultaneously with no extra effort.

In contrast to manual observations, automatic classification of species by random forest classification based on the output of BEMOVI allows users to quantify classification uncertainty. Classifications of individuals below a certain threshold could be flagged as "uncertain" or measures of classification error integrated in the statistical inference framework. A prerequisite for robust results of BEMOVI requires to compare its output against standard methods to quantify abundance or traits in a particular study system. Whereas we quantified the correspondence of manual and automatic counts, one could also have manually counted individuals on the videos recorded. This would allow to specifically disentangle the different sources of variability in automatic counts. Whereas we chose to compare results on the level of the microcosm, BEMOVI does not prevent users to choose different standards, especially if one is interested in integrating the sampling process in a state-space model; this is likely part of the necessary validation when applying BEMOVI (as any automated analysis approach) to a particular experimental system, that only the individual experimenter can perform on his/her data in the appropriate way given the system peculiarities.

Whereas the random forest algorithm provided low classification error in the smaller communities (three species), in the more complicated case of eight species with strong imbalance in the availability of individuals for training the algorithm, classification success was variable among classes. In particular, the complete misclassification of *P. aurelia*, although not unexpected due to the working of the random forest, warrants future improvements. Indeed, in random forest classification, under-represented classes may get lumped into the majority class if they are not easily distinguished, as is the case of *P. aurelia* and *P. caudatum*. However, note that this is not the case for *Blepharisma* for which only few individuals are available as well, but which gets classified very reliably. This behavior is due to the way the random forest classification works, which tries to minimize the impurities in the nodes (Cutler et al. 2007). Minority classes may overall cause negligible impurities (especially if imbalance is strong), although every case is misclassified. Random forest classification is only one among multiple classification methods with specific advantages and disadvantages. However, the automatic classification of species is not a feature of BEMOVI (i.e., there are no dedicated functions to perform this task), but rather a proof-of-concept that BEMOVI can provide the data allowing for species recognition, using random forest as an example of statistical analysis for such a goal. We suggest users dealing with these problems may use classification techniques better able to deal with large imbalances, for instance, Naive Bayes or support vector machines that take information about the relative abundance of classes into account.

Some limitations need to be considered when using BEMOVI. First, the tracking of individuals is performed in two dimensions, although the environment may be often three dimensional. Whereas this may be largely representative for organisms living on a plane (such as ground-dwelling insects for instance), for others such as aquatic organisms this simplification may be problematic for extrapolating the measured movement in the 3D environment. Several studies show, however, that 2D tracking successfully predicts spread rates even in higher dimensional systems (Giometto et al. 2014; Pennekamp 2014). Complex environments with many physical obstacles (e.g., debris particles in our medium) prevent reliable tracking in three dimensions, because individuals may be frequently invisible to one of the three required cameras. Nevertheless, rapid development of software and hardware will ultimately lead to systems performing 3D tracking (Dell et al. 2014).

Another limitation, which applies to video tracking in general, is the occurrence of occlusions, that is, when a particle collides with another particle (individual or debris) (Dell et al. 2014). This can result in errors and their propagation when identifying individuals (e.g., in studies of collective movement and social interactions in a swarm of animals). Some recently developed tracking algorithms such as the idTracker (Pérez-Escudero et al. 2014) or the Ctrax software (Branson et al. 2009) can deal with such situations using powerful "fingerprinting" techniques to keep track of individuals or including probabilistic models, which predict the position after the occlusion, and therefore may be able to maintain the individual identity. Due to the large numbers of individuals tracked simultaneously, the use of computationally intensive "fingerprinting" or manual verification are currently not suited to the goals of BEMOVI. However, given that the work flow is highly modular, another tracking software could easily replace the plug-in used and therefore extend for 3D tracking or having more power in maintaining individual identities. The same applies for the segmentation approach which is currently the dynamic difference image. BEMOVI will read and process data regardless of the segmentation method, as long it is provided in the format used by the package. So far, BEMOVI has no functionality to deal with clustering individuals (e.g., colonies). If these move as a cluster, they will be counted as one individual. Because BEMOVI was designed for communities of multiple species, splitting clusters automatically is a much harder problem than the single-species case, where size-based rules can be used (e.g., Pennekamp and Schtickzelle 2013). The watershed algorithm may have potential for improvement (Roerdink and Meijster 2000); however, if light conditions are heterogeneous and shapes of species differ considerably, the danger of oversplitting and splitting of non-clusters needs to be carefully evaluated. Extending and improving the functionality of BEMOVI is a long-term goal facilitated by the availability of the source code on the community coding platform Github. Finally, BEMOVI is not intended as a tool for microbiologists to screen for new species but rather to work either on communities in which species are known to differ in morphology and movement, or in natural communities where researchers want to gain information on size distributions and movement related traits (regardless of taxonomy).

Dell et al. (2014) conclude their review on automated video analysis in ecology with a call to developers. They ask for video analysis systems that are easy to use, do not require marking of individuals, are flexible to work with in a variety of experimental settings and with different organisms, allow tracking of a large number of individuals simultaneously, overcome significant data management issues, and are mostly automated. We believe BEMOVI is a promising step in this direction and will allow biologists to follow new and exciting research lines such as the effects of intraspecific and interindividual variation for ecological and evolutionary dynamics. Furthermore, although we tested utility for microbes, it is likely that BEMOVI will be useful for analyzing any objects moving against a relatively stationary background. For example, insects or birds on a surface could be tracked and analyzed, and it might even be possible to track and count birds flying in the sky or fish in the sea (although movement data would need to be treated with caution, given the 2D constraint of BEMOVI).

## Supporting Information

**Video S1.** Video footage of microcosm sample in counting chamber.

**Video S2.** Overlay video showing the individuals identified by BEMOVI and their respective trajectory labels.

**Video S3.** Overlay video showing the individuals of two ciliate species with different colour codes and their respective trajectory labels.

**Table S1.** Overview of traits/measures used in the automatic classification of species. **Figure S1.** Positive correlation between ciliate cell length measurements (collected from literature and online resources) and cell length measured by BEMOVI.
