# Supplementary material for: BEMOVI, software for extracting behavior and morphology from videos, illustrated with analyses of microbes
Source: Ecol Evol. 2015 Jun 4;5(13):2584–95. doi: 10.1002/ece3.1529 (PMC4523355; doi:10.1002/ece3.1529)
Supplement: Supplementary file 4 [file ece30005-2584-sd4.pdf]

SUPPLEMENTARY MATERIAL for

BEMOVI, software for extracting BEhaviour and MOrphology from VIdeos, illustrated with analyses of microbes

variable name	description	measure
area	cross-section of cell	mean and STD
perimeter	perimeter of cell	mean and STD
major	major axis of fitted ellipse i.e. length of cell	mean and STD
ar	aspect ratio of fitted ellipse $(\frac{majoraxis}{minoraxis})$	mean and STD
turning angle	relative change in direction compared to previous step	circular mean and STD
gross_disp	gross displacement	sum of individual step lengths
max_net	max net displacement	maximum of beeline distance between the starting position and each subsequent position
net_disp	net displacement	beeline distance between the first and the last position
net_speed	speed	$\frac{netdisplacement}{trajectoryduration}$
max_step	step length	maximum distance covered in one step i.e. between two frames
min_step	step length	minimum distance covered in one step i.e. between two frames
sd_step	step length	STD in step length
sd_gross_speed	gross movement speed	$\frac{grossdisplacement}{duration}$
max_gross_speed	maximum gross movement speed	maximum of $\frac{grossdisplacement}{duration}$
min_gross_speed	minimum gross movement speed	minimum of $\frac{grossdisplacement}{duration}$

Table S1: Overview of traits/measures used in the automatic classification of species. STD = standard deviation

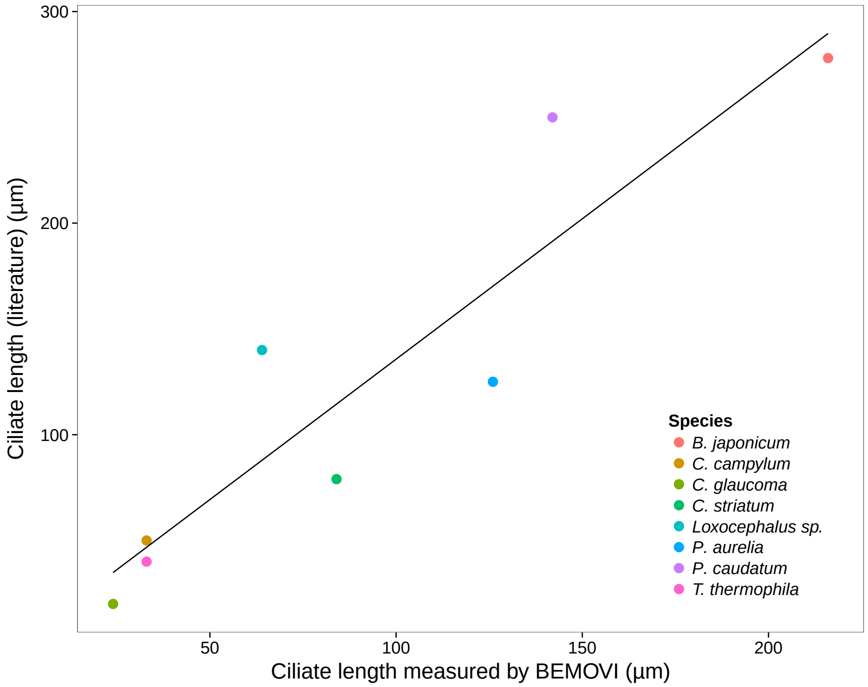

Figure S1: Positive correlation between ciliate cell length measurements (collected from literature and online resources) and cell length measured by BEMOVI.
